# A molecular model system based on human monoclonal allergen-specific IgE antibodies to study basophil activation

**DOI:** 10.1186/2045-7022-4-S2-P20

**Published:** 2014-03-17

**Authors:** Nazanin Najafi, Angelika Stoecklinger, Birgit Linhart, Christoph Madritsch, Christian Lupinek, Josef Thalhamer, Rudolf Valenta, Sabine Flicker

**Affiliations:** 1 Medical University of Vienna, Department of Pathophysiology and Allergy Research, Vienna, Austria; 2 University of Salzburg, Department of Molecular Biology, Salzburg, Austria

## Background & objective

The allergen-induced IgE-mediated effector cell activation is the key mechanism in acute allergic inflammation. The aim of this study was to produce human monoclonal IgE antibodies specific for major allergens and the corresponding allergens to study basophil activation.

## Methods

Monoclonal human IgE antibodies specific for the major birch pollen allergen Bet v 1 and for the major grass pollen allergen Phl p 5 and the corresponding allergens as well as a hybrid protein consisting of the Bet v 1 and Phl p 5 allergens were expressed. Using circular dichroism spectroscopy we compared the fold of the hybrid protein with that of the mixture of the two allergens. Rat basophil leukemia cells transfected with the human FcεRI receptor were then loaded with the monoclonal IgE antibodies or patients serum containing polyclonal allergen-specific IgE and then exposed to the allergens and the hybrid protein to study basophil activation.

## Results

We found that basophil activation was obtained with a mixture of two monoclonal IgE antibodies and the hybrid protein which increased in magnitude with rising concentrations of allergen-specific IgE antibodies. Thus increases in allergen-specific IgE lead to the release of larger amounts of mediators. When polyclonal IgE from allergic patients was used, basophil activation was obtained already with lower concentrations of the hybrid protein. An augmentation of the number of IgE epitopes thus increased the allergenic activity and thus the "potency" of the allergen. Preliminary titration experiments of the two monoclonal IgE antibodies also suggested that equimolar concentrations of the IgE antibodies with different specificity were most efficient in causing basophil activation, most likely due to the formation of large immune complexes.

## Conclusion

Results obtained in our molecular model for human basophil activation show that the levels of allergen-specific IgE dictate the extent of basophil degranulation and that the number of IgE epitopes determines the potency of an allergen to induce degranulation. The model should be useful to explore the factors determining allergen-induced basophil activation and to study therapeutic strategies controlling this event. This study was supported by grant P23318-B11 and in part by grants P23350-B11 and F4605 of the Austrian Science Fund (FWF).

